# Supplementary material for: Resolving the full spectrum of human genome variation using Linked-Reads
Source: Genome Res. 2019 Apr;29(4):635–45. doi: 10.1101/gr.234443.118 (PMC6442396; doi:10.1101/gr.234443.118)
Supplement: Supplemental Material [file supp_gr.234443.118_Supplemental_longranger-2.2.zip › longranger-2.2.tar.bz2!/lr2.2-public/lib/stan/cmdstan/stan/doc/api/html/quad__form__diag_8hpp_source.html]

Stan: src/stan/math/matrix/quad\_form\_diag.hpp Source File


|  |  |
| --- | --- |
|  | Stan  2.5.0  probability, sampling & optimization |


- Main Page
- Related Pages
- Namespaces
- Classes
- Files

- File List
- File Members

All Classes Namespaces Files Functions Variables Typedefs Enumerations Enumerator Friends Macros Pages

- src
- stan
- math
- matrix

quad\_form\_diag.hpp

Go to the documentation of this file.

1 #ifndef STAN\_\_MATH\_\_MATRIX\_\_QUAD\_FORM\_DIAG\_HPP

2 #define STAN\_\_MATH\_\_MATRIX\_\_QUAD\_FORM\_DIAG\_HPP

3

4 #include <stan/math/matrix/Eigen.hpp>

5 #include <boost/math/tools/promotion.hpp>

6 #include <stan/math/error\_handling/matrix/check\_square.hpp>

7 #include <stan/math/error\_handling/matrix/check\_vector.hpp>

8 #include <stan/math/error\_handling/check\_equal.hpp>

9

10 namespace stan {

11  namespace math {

12

13  using Eigen::Dynamic;

14  using Eigen::Matrix;

15  using boost::math::tools::promote\_args;

16

17  template <typename T1, typename T2, int R, int C>

18  inline Matrix

19  <typename promote\_args<T1,T2>::type, Dynamic, Dynamic>

20  quad\_form\_diag(const Matrix<T1, Dynamic, Dynamic>& mat,

21  const Matrix<T2, R, C>& vec) {

22  stan::math::check\_vector("quad\_form\_diag(%1%)", vec, "vec", (double\*)0);

23  stan::math::check\_square("quad\_form\_diag(%1%)",mat,"mat",(double\*)0);

24  int size = vec.size();

25  stan::math::check\_equal("quad\_form\_diag(%1%)",mat.rows(), size, "matrix size",

26  (double\*)0);

27  Matrix<typename promote\_args<T1,T2>::type, Dynamic, Dynamic>

28  result(size, size);

29  for (int i = 0; i < size; i++) {

30  result(i,i) = vec(i)\*vec(i)\*mat(i,i);

31  for (int j = i+1; j < size; ++j) {

32  typename promote\_args<T1,T2>::type temp = vec(i)\*vec(j);

33  result(j,i) = temp\*mat(j,i);

34  result(i,j) = temp\*mat(i,j);

35  }

36  }

37  return result;

38  }

39

40  }

41 }

42 #endif

stan::math::check\_equal

bool check\_equal(const char \*function, const T\_y &y, const T\_eq &eq, const char \*name, T\_result \*result)

**Definition:** check\_equal.hpp:57

stan::math::check\_vector

bool check\_vector(const char \*function, const Eigen::Matrix< T, R, C > &x, const char \*name, T\_result \*result)

**Definition:** check\_vector.hpp:14

check\_equal.hpp

stan::math::quad\_form\_diag

Matrix< typename promote\_args< T1, T2 >::type, Dynamic, Dynamic > quad\_form\_diag(const Matrix< T1, Dynamic, Dynamic > &mat, const Matrix< T2, R, C > &vec)

**Definition:** quad\_form\_diag.hpp:20

Eigen.hpp

check\_vector.hpp

stan::math::size

int size(const std::vector< T > &x)

**Definition:** size.hpp:11

stan::math::check\_square

bool check\_square(const char \*function, const Eigen::Matrix< T\_y, Eigen::Dynamic, Eigen::Dynamic > &y, const char \*name, T\_result \*result)

Return true if the specified matrix is square.

**Definition:** check\_square.hpp:24

check\_square.hpp


---

[ Stan Home Page ]

*© 2011–2014,
Stan Development Team.*
